# The rapidly changing treatment landscape of first-line advanced urothelial cancer (aUC) or metastatic urothelial cancer (mUC)

**DOI:** 10.37349/etat.2024.00258

**Published:** 2024-07-29

**Authors:** Minira Aslanova, Eun-Mi Yu, Jeanny B. Aragon-Ching

**Affiliations:** IRCCS Istituto Romagnolo per lo Studio dei Tumori (IRST) “Dino Amadori”, Italy; ^1^Division of Hematology and Oncology, Inova Schar Cancer Institute, Fairfax, VA 22031, USA; ^2^GU Medical Oncology, Inova Schar Cancer Institute, Fairfax, VA 22031, USA

**Keywords:** Cisplatin-eligible, avelumab maintenance, enfortumab vedotin, pembrolizumab, nivolumab

## Abstract

The landscape of treatment for first-line therapy in advanced urothelial cancer (aUC) and metastatic urothelial cancer (mUC) has rapidly changed in the last year alone. Maintenance avelumab remains a viable treatment option for many patients across the globe for those who have responded or have achieved stable disease after platinum-based chemotherapy. However, the recent FDA approvals based on EV-302 for enfortumab vedotin (EV) and pembrolizumab, as well as CheckMate-904 with gemcitabine and cisplatin with nivolumab (GC+N) followed by maintenance nivolumab have left clinicians with the complicated decision of determining which regimen is most appropriate for their individual patients with untreated aUC. This commentary highlights the key trials that have set the standard-of-care for front-line aUC treatment and suggestions for choosing different regimens for the appropriate patient.

## Introduction

**Figure 1:**
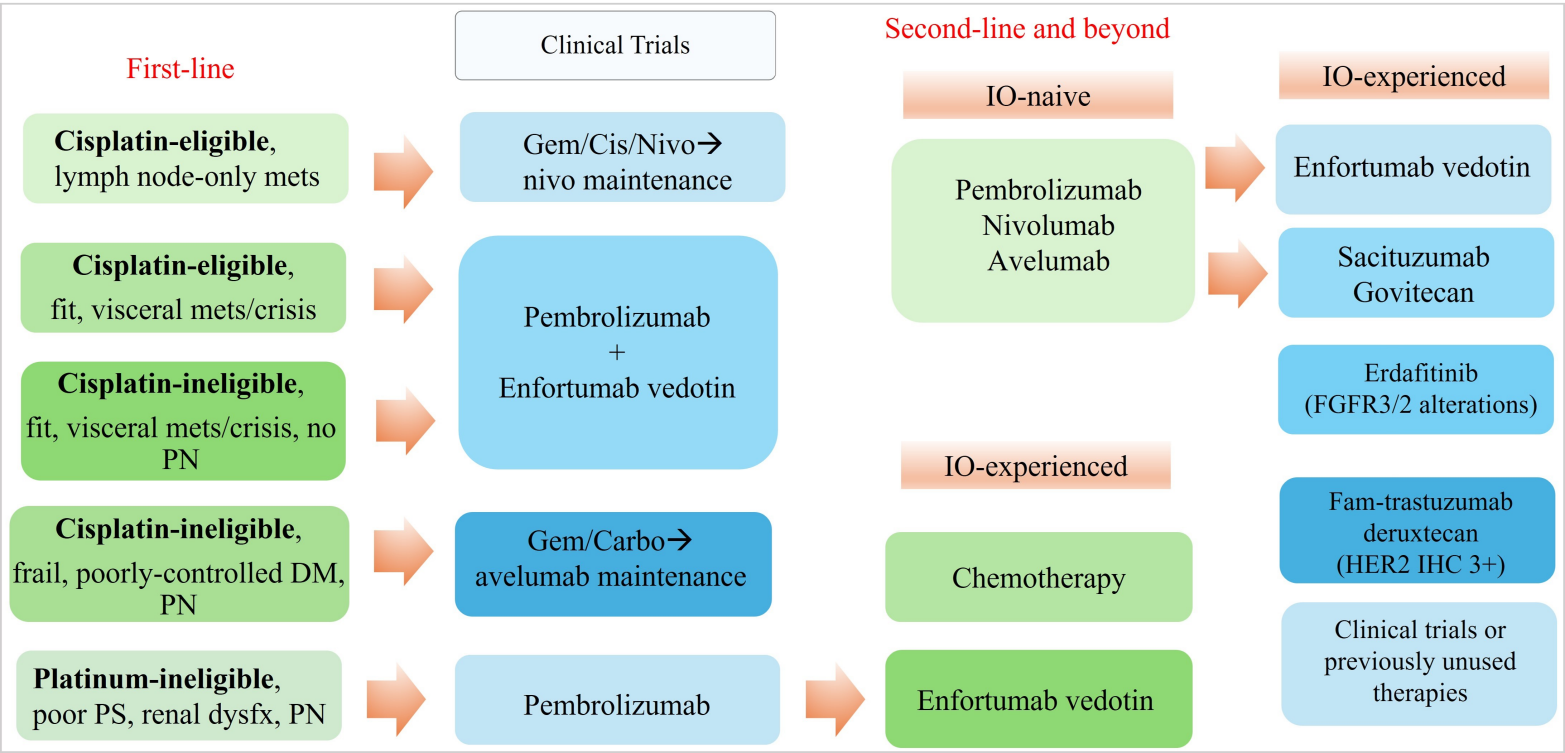
The proposed treatment paradigm for metastatic urothelial cancer (mUC). Carbo: carboplatin; cis: cisplatin; DM: diabetes mellitus; FGFR: fibroblast growth factor receptor; gem: gemcitabine; HER2: human epidermal growth factor receptor 2; IHC: immunohistochemistry; IO: immuno-oncology; mets: metastasis; nivo: nivolumab; PN: peripheral neuropathy; mets: metastases; dysfx: dysfunction; PS: performance status

Bladder cancer is the 4th most common cancer in men with an estimated incidence of 63,070 patients diagnosed in 2024 alone in the United States [1]. The treatment of advanced urothelial cancer (aUC) involved the use of platinum-based chemotherapy (PBC) in the last few decades until the emergence of immuno-oncology (IO) drugs of which five were initially available for the treatment of aUC (see Figure 1). In 2017, anti-PD-L1 monoclonal antibody drugs atezolizumab [2] and avelumab [3], and anti-PD-1 monoclonal antibody agents pembrolizumab [4], nivolumab [5], and durvalumab [6] were available for use in aUC. Single-agent pembrolizumab and atezolizumab were frequently used until atezolizumab was voluntarily withdrawn by Genentech after the results of the IMvigor-211 trial [7] and the FDA limited use of pembrolizumab to patients with aUC patients who are platinum-ineligible [8]. Durvalumab was also withdrawn from use in aUC based on the negative DANUBE trial [9]. Responses to IO monotherapy were modest in the first and second-line setting, so efforts to improve responses to IO monotherapy eventually led to the practice-changing JAVELIN Bladder 100 trial in 2020 [10]. Switch maintenance therapy with avelumab was found to improve overall survival (OS) and progression-free survival (PFS) in patients who achieve at least stable disease (SD) after first-line PBC, which changed the standard-of-care in this patient population. Erdafitinib, a novel tyrosine kinase inhibitor, was approved in 2019 for patients with previously-treated aUC whose tumors harbor certain fibroblast growth factor receptor 2 (FGFR2) or FGFR3 alterations [11]. The phase III confirmatory trial, THOR, showed improvement in OS and PFS with erdafitinib vs. chemotherapy in patients with aUC previously-treated with IO therapy harboring certain FGFR2/3 alterations [12]. Other targeted agents used in aUC include antibody-drug conjugates (ADCs) such as enfortumab vedotin (EV) and sacituzumab govitecan (SG). EV targets Nectin-4 and it was granted accelerated approval by the FDA in 2019 based on an objective response rate (ORR) of 44% in the EV-201 trial which included cisplatin-ineligible patients previously treated with IO therapy [13]. SG is an ADC that targets Trop-2 and it was granted accelerated approval in 2021 based on the TROPHY-U01, a 112-patient single-arm trial that showed an ORR of 27.7% with the use of SG in patients previously treated with PBC and IO [14].

Recently, two major first-line trials, EV-302 [15] and CheckMate-901 [16] led to the approval of two additional treatment regimens for aUC [17]. The NCCN updated its treatment guidelines and continues to offer recommendations primarily based on cisplatin eligibility. Cisplatin-ineligibility is widely defined by the Galsky criteria which date back to 2011: (a) Eastern Cooperative Oncology Group performance status of ≥ 2; (b) creatinine clearance < 60 mL/min; (c) grade ≥ 2 hearing loss, grade ≥ 2 neuropathy; or (d) New York Heart Association Class III heart failure [18]. Preferred first-line regimens include PBC followed by maintenance avelumab, EV plus pembrolizumab (EV+P), and gemcitabine and cisplatin with nivolumab (GC+N) followed by nivolumab maintenance. Cisplatin-ineligible patients may consider PBC with carboplatin followed by maintenance avelumab or EV+P in the first-line setting. The details of these landmark studies are summarized in Table 1 and are further discussed herein.

**Table 1:** Comparison between phase III front-line UC trials

**Trial**	**Experimental arm vs. standard arm**	**Primary endpoint**	**Secondary endpoints**	**Toxicity**	**US FDA approval**
JAVELIN Bladder 100 [19]	Gem/cis or carbo followed by avelumab + BSC vs. BSC	Updated mOS: 23.8 mos (Ave) vs. 15 mos (BSC); OS at 1 year: 71.3% (Ave) vs. 58.4% (BSC)	mPFS: 5.5 mos (Ave) vs. 2.1 mos (BSC); updated 2-year PFS: 23.4% (Ave) vs. 7.1% (BSC); trAE: 98.3% pts (Ave group) vs. 77.0% pts (BSC); at eligibility: CR 25.7%; PR = 46.6%; SD = 27.7%	Grade >/= 3: Ave (47.4%) vs. BSC (77.7%); Ave included: UTI (4.4%), anemia (3.8%), hematuria (1.7%), fatigue (1.7%), back pain (1.2%), vomiting (1.2%)	June 30, 2020: Ave maintenance treatment of pts with la or mUC that has not progressed with first-line platinum-containing chemotherapy
EV-302 [15]	EV+P vs. gem/cis or carbo	mOS: 31.5 mos (EV+P group) vs. 16.1 mos (chemotherapy group); PFS: 12.5 mos (EV+P group) vs. 6.3 mos (chemotherapy group)	ORR: 67.7% (CR = 29.1%) in the EV+P vs. 44.4% (CR = 12.5%) in the chemotherapy group; TTP: 14.2 mos (EV+P group) vs. 10.0 mos (chemotherapy group)	Grade >/= 3 trAE: 55.9% with EV+P vs. 69.5% with chemotherapy; EV+P grade >/= 3 included: rash (7.7%), hyperglycemia (5.0%), neutropenia (4.8%)	December 15, 2023: EV+P for pts with locally advanced or mUC
CheckMate 901 (sub-study) [16]	Gem/cis + nivo vs. gem/cis	mOS: 21.7 mos (GC + nivo) vs. 18.9 mos (gem/cis); PFS at 12 mos: 34.2% (GC + nivo) vs. 21.8% (gem/cis)	ORR: 57.6% (CR = 21.7%) GC + nivo vs. 43.1% (CR = 11.8%) GC; OS and PFS in PD-L1 expression of >/= 1% favored GC + nivo (HR: 0.75, 95% CI: 0.53 to 1.06) over GC group (HR: 0.60, 95% CI: 0.41 to 0.81)	Grade >/= 3 AE: 61.8% of gem/cis + nivo group vs. 51.7% in gem/cis group; trAE with gem/cis + nivo were anemia (57.2%), nausea (46.7%), and neutropenia (30.6%)	March 6, 2024: GC + nivo for first-line treatment of pts with unresectable or mUC

Ave: avelumab; cis: cisplatin; carbo: carboplatin; gem: gemcitabine; nivo: nivolumab; EV: enfortumab vedotin; EV+P: enfortumab vedotin plus pembrolizumab; BSC: best supportive care; PFS: progression-free survival; OS: overall survival; mOS: median overall survival; mos: months; ORR: objective response rates; TPP: time to pain progression; mUC: metastatic urothelial carcinoma; trAE: treatment-related adverse events; la: locally advanced; AE: adverse events; GC: gemcitabine/cisplatin; UTI: urinary tract infection; TTP: time to progression; HR: hazard ratios; CI: confidence intervals; pts: patients; CR: complete response; SD: stable disease; PR: partial response; PD-L1: programmed death ligand-1

## JAVELIN Bladder 100 trial

JAVELIN Bladder 100 enrolled 700 patients with unresectable aUC who received four to six cycles of PBC (gemcitabine plus cisplatin or carboplatin) and did not have disease progression during or upon completion of PBC [10]. Patients were randomized to receive avelumab maintenance plus best supportive care (BSC) or BSC only starting within 4–10 weeks of their last dose of PBC. The primary end point for the trial was OS assessed in the overall population and separately in patients with tumors positive for PD-L1 expression. Key secondary endpoints of the trial were PFS and safety.

Maintenance avelumab significantly prolonged OS when added to BSC compared to BSC alone. The trial met its primary endpoint by prolonging the 1-year OS rates in the avelumab group at 71.3% vs. 58.4% in the BSC alone arm. Two-year OS rates were 49.8% in the avelumab arm vs. 38.4% in the control arm. OS was also noted to be significantly prolonged in the PD-L1 positive population, with improved survival at 1 year of 79.1% in the avelumab group vs. 60.4% in the BSC group [19]. In a posthoc exploratory analysis, median OS (mOS) from the start of chemotherapy was 29.7 months in the avelumab group vs. 20.5 months in the BSC-only group [hazard ratios (HR) 0.77, 95% confidence intervals (CI): 0.64–0.92], with benefits across intervals of chemotherapy start [20]. Of note, most patients in the BSC-only group (72%) went on to receive second-line therapy, most often with IO therapy. Despite the frequent use of IO therapy upon recurrence in the control group, patients receiving avelumab maintenance group still had an improved OS compared to patients in the control arm.

The trial met its secondary endpoint of improved PFS although the median PFS (mPFS) was modest at 5.5 months (95% CI: 4.2 to 7.2) in the avelumab arm vs. 2.1 months (95% CI: 1.9 to 3.0) in the BSC arm. In an updated analysis, the 2-year PFS rate was 23.4% in the avelumab arm vs. 7.1% in the control arm [19].

The incidence of adverse events (AEs) was higher in the avelumab maintenance group vs. the control group. AEs of any grade occurred in about 98.0% of patients in the avelumab group (47.4% of grade 3 or higher AEs) vs. 77.7% of patients in the BSC-only group (25.2% of grade 3 or higher AEs).

The trial met its secondary endpoint of improved PFS although the median PFS (mPFS) was modest at 5.5 months (95% CI: 4.2 to 7.2) in the avelumab arm vs. 2.1 months (95% CI: 1.9 to 3.0) in the BSC arm. In an updated analysis, the 2-year PFS rate was 23.4% in the avelumab arm vs. 7.1% in the control arm [19].

The incidence of AEs was higher in the avelumab maintenance group vs. the control group. AEs of any grade occurred in about 98.0% of patients in the avelumab group (47.4% of grade 3 or higher AEs) vs. 77.7% of patients in the BSC-only group (25.2% of grade 3 or higher AEs).

With these practice-changing results, the use of switch maintenance avelumab quickly became standard-of-care in 2020. The benefits of avelumab maintenance over BSC alone appear to be maintained in long-term follow-up analyses [21]. Moreover, recent patient-reported outcomes show no detrimental impacts on quality of life or new safety concerns [22].

## EV-302 trial

EV-302 was a phase III trial of EV, a Nectin-4-directed ADC combined with pembrolizumab, a PD-1 inhibitor vs. PBC in patients with previously untreated aUC. A total of 886 patients underwent 1:1 randomization to either receive EV+P or gemcitabine + cisplatin or carboplatin (based on Galsky cisplatin-ineligibility criteria). Patients were stratified based on PD-L1 expression and the presence of liver metastases. Clinical characteristics of the patients in the two treatment arms were well-balanced, with a median age of 69 years, but only 3.4% of patients had Eastern Cooperative Oncology Group Performance Status (ECOG PS) 2 in the EV+P arm.

Treatment was continued until disease progression, the start of subsequent therapy, unacceptable toxicities, or completion of the maximum number of treatment cycles. The maximum number of cycles for patients in the PBC arm was 6. In those receiving EV+P, there was no maximum set for EV but the maximum number of cycles for pembrolizumab was 35 cycles.

The primary endpoints of the study were OS and PFS, with key secondary endpoints including safety, duration of response (DoR), time to pain progression, and overall response. mPFS was 12.5 months (95% CI: 10.4 to 16.6) in the EV+P arm vs. 6.3 months (95% CI: 6.2 to 6.5) in the PBC arm. mOS was 31.5 months in the EV+P group vs. 16.1 months in the chemotherapy arm (HR: 0.47, 95% CI: 0.38 to 0.58, *P* < 0.00001). Consistent PFS and OS benefits were observed across the intention-to-treat (ITT) population and all prespecified subgroups including those defined by cisplatin eligibility, PD-L1 expression status, and presence of visceral/liver/lymph node metastases.

Overall response favored the EV+P arm vs. the chemotherapy arm with a 67.7% (95% CI: 63.1 to 72.1) vs. 44.4% (95% CI: 39.7 to 49.2, *P* < 0.001) response rates, respectively. Complete response (CR) was observed in 29.1% vs. 12.5% in the EV+P and PBC arms, respectively. Median DoR was not met in the EV+P group but was 7 months in the PBC arm. Remission rates at 12 and 18 months were 67.3% and 59.6% with EV+P vs. 35.2% and 19.3% with PBC. Around 70% vs. 32% of patients in the EV+P and PBC arms received subsequent therapy for aUC, respectively [15].

Reported AEs were consistent with the known safety profiles of each treatment arm. Grade 3 and higher treatment-related AEs occurred in 55.9% of patients in the EV+P group, and 69.5% in the chemotherapy group. However, serious AEs occurred in 27.7% of patients in the EV+P group compared to 19.6% in the chemotherapy group. Specific AEs that occurred significantly more often in the EV+P arm included peripheral neuropathy, alopecia, diarrhea, hyperglycemia, and rash [15]. Results of the EV-302 trial support EV+P as a new standard-of-care option for the first-line treatment of aUC.

## CheckMate-901 trial

CheckMate-901 was a randomized open-label phase III trial completed in two parts in untreated aUC patients. The first part of the trial evaluated the addition of nivolumab, a PD-1 inhibitor, to cisplatin plus gemcitabine (GC+N). The second part compared nivolumab, a PD-1 inhibitor, plus ipilimumab, a cytotoxic T-lymphocyte antigen-4 (CTLA-4) inhibitor, with PBC. The primary endpoints of OS and PFS were evaluated in all patients, as well as in prespecified subgroups. Key secondary endpoints included OS and PFS in patients with tumor cell PD-L1 expression of ≥ 1%. The results of the first part of the trial, discussed herein, were published in October 2023 [16].

While nivolumab/ipilimumab failed to meet the OS endpoint over PBC in the first-line treatment of aUC patients with PD-L1expression of ≥ 1% [23], the results of the first part of the CheckMate-901 trial evaluating GC+N vs. GC alone for cisplatin-eligible aUC patients were positive. 608 patients underwent 1:1 randomization to receive GC+N every 3 weeks for up to six cycles, followed by nivolumab every 4 weeks for a maximum of 2 years, or to receive GC alone every 3 weeks for up to 6 cycles [16]. Clinical characteristics were well-balanced between the two groups, with a median patient age of 65 years. Median follow-up for the study was 33.6 months and the median duration of study therapy was 7.4 months for the GC+N group compared to 3.7 months for the GC group. OS was significantly longer in the GC+N group (HR of 0.78; 95% CI: 0.63 to 0.96; *P* = 0.02) with mOS of 21.7 months (95% CI: 18.6 to 26.4) vs. 18.9 months (95% CI: 14.7 to 22.4) in the GC group. The mPFS per Blinded Independent Central Review was 7.9 months in the N+GC group compared to 7.6 months in the GC group, with HR of 0.72 (95% CI: 0.59 to 0.88, *P* = 0.001). Subgroup analysis of OS and PFS also favored GC+N over chemotherapy only, including populations with PD-L1 expression ≥ 1%. ORR was 57.6% in the GC+N arm and 43.1% in the GC arm. Safety outcomes were consistent with previously reported AEs for each of the drugs used. Grade 3 and higher treatment-related AEs occurred in 61.8% of receiving GC+N and in 51.7% of patients receiving GC. Health-related quality-of-life outcomes were stable based on the European Organisation for Research and Treatment of Cancer 30-item core instrument (EORTC QLQ-C30) questionnaires completed by study patients.

## Choosing a first-line combination regimen

CheckMate-901 highlighted the benefits of concurrent and maintenance IO therapy with standard GC chemotherapy in cisplatin-eligible aUC patients. With multiple treatment regimens to consider in untreated aUC patients, clinicians need to understand the complexities and nuances of each trial to engage in thoughtful decision-making with individual patients. With JAVELIN Bladder 100, the treatment paradigm for aUC first shifted from first-line PBC up to 6 cycles, surveillance, then second-line therapy on disease progression to the routine use of maintenance avelumab upon completion of PBC when at least SD is achieved. While we acknowledge the positive results of the CheckMate-901 and EV-302 trials, a shared limitation of both trials was that the control arms primarily consisted of PBC only without maintenance avelumab which became standard practice in 2020.

KEYNOTE-361 [24] and IMVigor-130 [25] were two other trials that evaluated the concurrent use of chemotherapy and IO therapy, but they failed to show the superiority of concurrent chemotherapy and immunotherapy over chemotherapy alone. However, a key difference with these negative studies was that they included cisplatin-ineligible patients who received carboplatin instead of cisplatin. In KEYNOTE-361, mPFS was similar between the IO combination arm at 8.3 months and the PBC arm at 7.1 months (HR 0.78, 95% CI: 0.65–0.93, *P* = 0.0033). There was a trend towards OS improvement with adding IO to PBC, but it did not achieve statistical significance. There was no benefit to adding pembrolizumab even among the patients receiving cisplatin-based chemotherapy. The positive results reported in the CheckMate-901 trial challenged the findings in KEYNOTE-361, but in contrast to the latter trial, all patients in CheckMate-901 received cisplatin and gemcitabine. In KEYNOTE-361, 56% and 44% of patients in the chemotherapy-containing arms received carboplatin and cisplatin, respectively. Similarly, most of the patients in the chemotherapy arms of IMVigor-130 received carboplatin instead of cisplatin [25].

Acknowledging the differences between the patient populations and limitations of cross-trial comparisons, the mOS in JAVELIN Bladder 100 and CheckMate-901 appear to be comparable in the IO-containing arms of each trial, 23.8 vs. 21.7 months, respectively, based on an updated report on JAVELIN Bladder 100 [19]. Although the reported ORR is vastly higher at 57.6% in the IO combination arm of the CheckMate-901 trial compared to 14.3% in the maintenance avelumab arm of the JAVELIN Bladder 100 trial, it is noted that response was measured from when patients were randomized to maintenance avelumab + BSC or BSC only after completion of PBC in the JAVELIN Bladder 100 trial. ORR was measured from the start of first-line GC+N or GC in the CheckMate-901 trial, so it included tumor responses achieved during chemotherapy. CR rates in the CheckMate-901 and JAVELIN Bladder 100 trials were 21.7% and 25.7%, respectively (see Table 1), though only 7.1% at the beginning of maintenance avelumab, suggestive of a synergistic effect when IO is combined with chemotherapy upfront though again the responses from JAVELIN were in large part due to the upfront chemotherapy responses so no patients with progressive disease as a best response would have been eligible. However, cisplatin’s superiority over carboplatin is well-known and most patients in the CheckMate-901 trial received cisplatin. Median duration of any response was 9.5 months with GC+N in the CheckMate-901 trial, but 28.4 months for maintenance avelumab (but among all randomly assigned patients on trial, the restricted mean DoR was longer by just 2.8 months compared to the BSC only arm) in the JAVELIN Bladder 100 trial. More specifically, the median duration of CR was 37.1 months with patients receiving GC+N in the CheckMate-901 trial, suggesting an association between depth of response and durability of response. Based on subgroup analyses of OS in these trials, patients with liver metastases may fare better with the GC+N regimen compared to PBC followed by maintenance avelumab as patients with liver metastases seemed to benefit less from maintenance avelumab compared to those without liver metastases. In a more recent comprehensive clinical subgroup analysis of the JAVELIN Bladder 100 trial, the HR for OS in the avelumab arm in patients with liver and lung metastases were 0.92 and 0.88, respectively. This is in contrast to lower HR for OS of 0.65 and 0.63, in avelumab-receiving patients without liver and without lung metastases, respectively [21]. Likewise, patients with upper tract UC (UTUC) tended to have less OS benefit with maintenance avelumab compared to those with lower tract primaries, though this is in keeping with what is known about the clinical behavior of UTUCs. Furthermore, certain subgroup outcomes including that of lymph node-only subsets may exhibit durable responses in CheckMate-901. Unfortunately, the question of which treatment approach would best suit patients with UTUC may not be addressed by CheckMate-901 given the relatively smaller proportion of these patients in this trial. On review of safety outcomes in each of these trials, the rate of grade 3 or higher AE was slightly higher with GC+N compared to patients receiving PBC followed by avelumab, again acknowledging the limitations of cross-trial comparison. Therefore, one might make a case for sequential chemotherapy and IO therapy in patients who are cisplatin-ineligible and/or otherwise perceived to be at higher risk of developing treatment toxicity. Another consideration is that maintenance avelumab is continued indefinitely until development of unacceptable toxicity or progression of the disease, per the JAVELIN Bladder 100 protocol. With CheckMate-901, protocol dictates stopping nivolumab after two years of therapy which clinicians and patients may be reluctant to follow in the real-world setting in the absence of longer-term data if nivolumab is otherwise effective and tolerated well.

Lastly, EV+P is another first-line option based on the unprecedented results of the EV-302 trial including in patients with visceral metastases and UTUC. Therefore, EV+P would be a viable treatment option for most patients with aUC especially those who are at the highest risk for visceral crisis. The mOS was of 31.5 months vs. 16.1 months (HR 0.47; 95% CI: 0.38 to 0.58; *P* < 0.001). While PFS was impressive at 12.5 months vs. 6.3 months, with a CR rate of 29.1% in those who received EV+P, treatment-related AEs were not trivial. Half of the patients receiving EV+P experienced peripheral sensory neuropathy compared to 9.9% in the chemotherapy arm. Other toxicities including alopecia, diarrhea, hyperglycemia, and rash were prominent in the EV+P arm compared to the PBC arm. Myelosuppression occurred less frequently with EV+P than with PBC. One strength of EV-302 over CheckMate-901 was that about 30.4% of patients in the PBC arm received maintenance avelumab.

**Figure 2:**
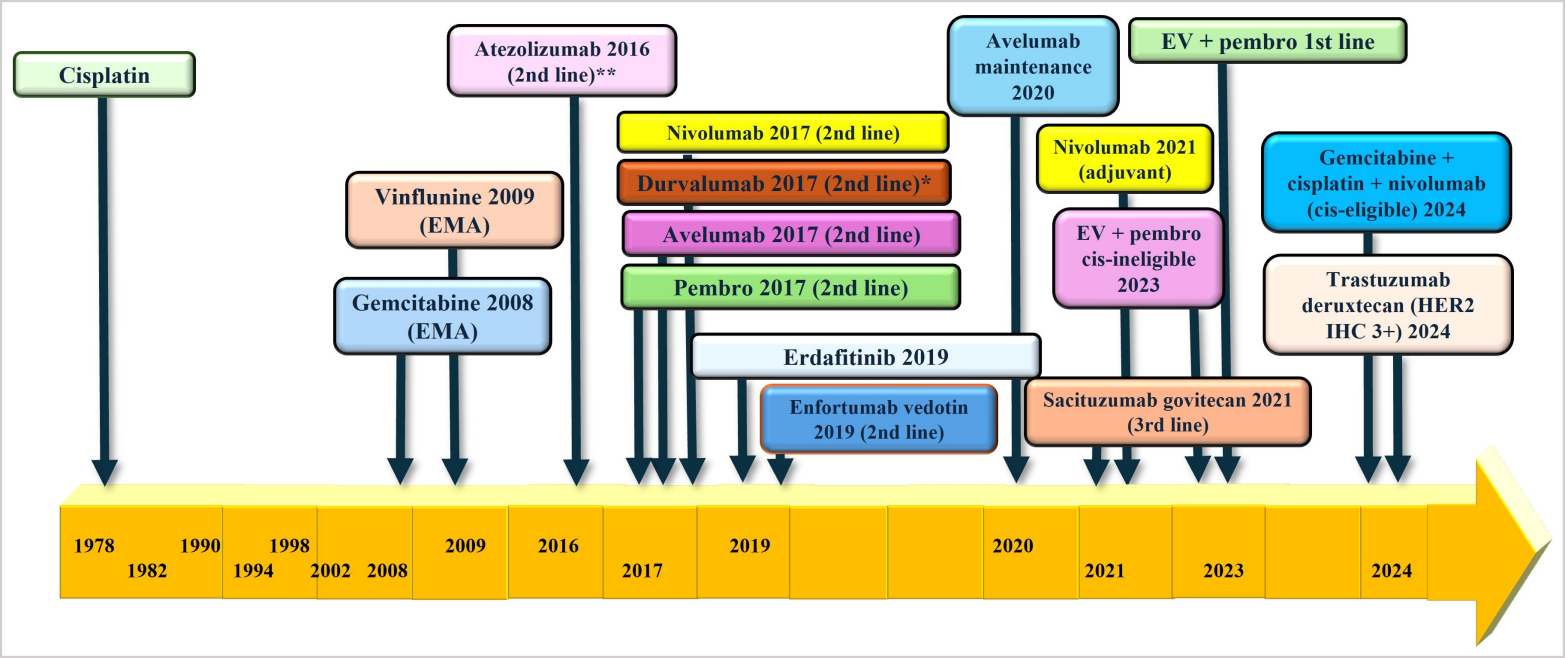
Regulatory approval for bladder cancer. EMA: European Medicine Agency; pembro: pembrolizumab; EV: enfortumab vedotin; cis: cisplatin; HER2: human epidermal growth factor receptor 2; IHC: immunohistochemistry. * voluntary withdrawal from AstraZeneca in 2021; ** voluntary withdrawal from Roche in 2022

In summary, for most treatment-naive aUC, EV+P is a strong consideration for most patients with aUC especially those who are fit and at risk for visceral crisis (liver metastases, high-burden of disease, etc.). Patients who may have lymph-node-only disease and considered cisplatin-eligible would be candidates for GC+N and conceivably, some may even proceed with consolidative cystectomy. However, for cisplatin-ineligible patients, carboplatin + gemcitabine followed by maintenance avelumab would still be a consideration though understanding that real-world implementation had been challenging due to high attrition from progressive disease. EV+P should be considered for cisplatin-ineligible patients who do not have significant pre-existing neuropathy. However, patients considered cisplatin-ineligible or platinum-ineligible with significant pre-existing neuropathy or frailty and comorbidities would also poorly tolerate EV+P, hence pembrolizumab monotherapy may be most appropriate. As longer-term data on these trials emerge, we hope that first-line recommendations for aUC patients can be further individualized (see Figure 2) over time to delay second-line therapy and maximize life expectancy while maintaining quality-of-life.

## Abbreviations

ADCs: antibody-drug conjugates
AEs: adverse events
aUC: advanced urothelial cancer
BSC: best supportive care
CR: complete response
DoR: duration of response
EV+P: enfortumab vedotin plus pembrolizumab
FGFR2/3: fibroblast growth factor receptor 2/3
GC+N: gemcitabine and cisplatin with nivolumab
HR: hazard ratio
IO: immuno-oncology
mPFS: median progression-free survival
ORR: objective response rate
OS: overall survival
PBC: platinum-based chemotherapy
PD-L1: programmed death ligand-1
PFS: progression-free survival
SG: sacituzumab govitecan
UTUC: upper tract urothelial cancer

## Declarations

### Copyright

© The author (s) 2024.

## References

[1] Siegel RL, Giaquinto AN, Jemal A (2024). Cancer statistics, 2024. CA Cancer J Clin..

[2] Balar AV, Galsky MD, Rosenberg JE, Powles T, Petrylak DP, Bellmunt J, IMvigor210 Study Group (2017). Atezolizumab as first-line treatment in cisplatin-ineligible patients with locally advanced and metastatic urothelial carcinoma: a single-arm, multicentre, phase 2 trial. Lancet..

[3] Patel MR, Ellerton J, Infante JR, Agrawal M, Gordon M, Aljumaily R (2018). Avelumab in metastatic urothelial carcinoma after platinum failure (JAVELIN Solid Tumor): pooled results from two expansion cohorts of an open-label, phase 1 trial. Lancet Oncol..

[4] Balar AV, Castellano D, O’Donnell PH, Grivas P, Vuky J, Powles T (2017). First-line pembrolizumab in cisplatin-ineligible patients with locally advanced and unresectable or metastatic urothelial cancer (KEYNOTE-052): a multicentre, single-arm, phase 2 study. Lancet Oncol..

[5] Sharma P, Retz M, Siefker-Radtke A, Baron A, Necchi A, Bedke J (2017). Nivolumab in metastatic urothelial carcinoma after platinum therapy (CheckMate 275): a multicentre, single-arm, phase 2 trial. Lancet Oncol..

[6] Powles T, O’Donnell PH, Massard C, Arkenau H, Friedlander TW, Hoimes CJ (2017). Efficacy and Safety of Durvalumab in Locally Advanced or Metastatic Urothelial Carcinoma: Updated Results From a Phase 1/2 Open-label Study. JAMA Oncol..

[7] Powles T, Durán I, Heijden MSvd, Loriot Y, Vogelzang NJ, Giorgi UD (2018). Atezolizumab versus chemotherapy in patients with platinum-treated locally advanced or metastatic urothelial carcinoma (IMvigor211): a multicentre, open-label, phase 3 randomised controlled trial. Lancet..

[8] FDA limits the use of Tecentriq and Keytruda for some urothelial cancer patients [Internet]. https://www.fda.gov/drugs/resources-information-approved-drugs/fda-limits-use-tecentriq-and-keytruda-some-urothelial-cancer-patients.

[9] Powles T, Heijden MSvd, Castellano D, Galsky MD, Loriot Y, Petrylak DP, DANUBE study investigators (2020). Durvalumab alone and durvalumab plus tremelimumab versus chemotherapy in previously untreated patients with unresectable, locally advanced or metastatic urothelial carcinoma (DANUBE): a randomised, open-label, multicentre, phase 3 trial. Lancet Oncol..

[10] Powles T, Park SH, Voog E, Caserta C, Valderrama BP, Gurney H (2020). Avelumab Maintenance Therapy for Advanced or Metastatic Urothelial Carcinoma. N Engl J Med..

[11] Loriot Y, Necchi A, Park SH, Garcia-Donas J, Huddart R, Burgess E, BLC2001 Study Group (2019). Erdafitinib in Locally Advanced or Metastatic Urothelial Carcinoma. N Engl J Med..

[12] Loriot Y, Matsubara N, Park SH, Huddart RA, Burgess EF, Houede N, THOR Cohort 1 Investigators (2023). Erdafitinib or Chemotherapy in Advanced or Metastatic Urothelial Carcinoma. N Engl J Med.

[13] Rosenberg JE, O’Donnell PH, Balar AV, McGregor BA, Heath EI, Yu EY (2019). Pivotal Trial of Enfortumab Vedotin in Urothelial Carcinoma After Platinum and Anti-Programmed Death 1/Programmed Death Ligand 1 Therapy. J Clin Oncol..

[14] Tagawa ST, Balar AV, Petrylak DP, Kalebasty AR, Loriot Y, Fléchon A (2021). TROPHY-U-01: A Phase II Open-Label Study of Sacituzumab Govitecan in Patients With Metastatic Urothelial Carcinoma Progressing After Platinum-Based Chemotherapy and Checkpoint Inhibitors. J Clin Oncol..

[15] Powles T, Valderrama BP, Gupta S, Bedke J, Kikuchi E, Hoffman-Censits J, EV-302 Trial Investigators (2024). Enfortumab Vedotin and Pembrolizumab in Untreated Advanced Urothelial Cancer. N Engl J Med..

[16] Heijden MSvd, Sonpavde G, Powles T, Necchi A, Burotto M, Schenker M, CheckMate 901 Trial Investigators (2023). Nivolumab plus Gemcitabine-Cisplatin in Advanced Urothelial Carcinoma. N Engl J Med..

[17] NCCN Clinical Practice Guidelines in Oncology (NCCN Guidelines^®^)-Bladder Cancer [Internet]. https://www.nccn.org/professionals/physician_gls/pdf/bladder.pdf.

[18] Galsky MD, Hahn NM, Rosenberg J, Sonpavde G, Hutson T, Oh WK (2011). Treatment of patients with metastatic urothelial cancer “unfit” for Cisplatin-based chemotherapy. J Clin Oncol..

[19] Powles T, Park SH, Caserta C, Valderrama BP, Gurney H, Ullén A (2023). Avelumab First-Line Maintenance for Advanced Urothelial Carcinoma: Results From the JAVELIN Bladder 100 Trial After ≥ 2 Years of Follow-Up. J Clin Oncol..

[20] Sridhar SS, Powles T, Climent Duran MA, Park SH, Massari F, Thiery-Vuillemin A (2024). Avelumab First-line Maintenance for Advanced Urothelial Carcinoma: Analysis from JAVELIN Bladder 100 by Duration of First-line Chemotherapy and Interval Before Maintenance. Eur Urol.

[21] Grivas P, Park SH, Voog E, Caserta C, Gurney H, Bellmunt J (2023). Avelumab First-line Maintenance Therapy for Advanced Urothelial Carcinoma: Comprehensive Clinical Subgroup Analyses from the JAVELIN Bladder 100 Phase 3 Trial. Eur Urol..

[22] Grivas P, Kopyltsov E, Su P, Parnis FX, Park SH, Yamamoto Y (2023). Patient-reported Outcomes from JAVELIN Bladder 100: Avelumab First-line Maintenance Plus Best Supportive Care Versus Best Supportive Care Alone for Advanced Urothelial Carcinoma. Eur Urol..

[23] Bristol Myers Squibb provides update on CheckMate -901 trial evaluating Opdivo (nivolumab) plus Yervoy (ipilimumab) as first-line treatment for patients with unresectable or metastatic urothelial carcinoma [Internet]. https://news.bms.com/news/corporate-financial/2022/Bristol-Myers-Squibb-Provides-Update-on-CheckMate--901-Trial-Evaluating-Opdivo-nivolumab-Plus-Yervoy-ipilimumab-as-First-Line-Treatment-for-Patients-with-Unresectable-or-Metastatic-Urothelial-Carcinoma/default.aspx.

[24] Powles T, Csőszi T, Özgüroğlu M, Matsubara N, Géczi L, Cheng SY, KEYNOTE-361 Investigators (2021). Pembrolizumab alone or combined with chemotherapy versus chemotherapy as first-line therapy for advanced urothelial carcinoma (KEYNOTE-361): a randomised, open-label, phase 3 trial. Lancet Oncol..

[25] Grande E, Arranz JA, De Santis M, Bamias A, Kikuchi E, Del Muro XG (2024). Atezolizumab plus chemotherapy versus placebo plus chemotherapy in untreated locally advanced or metastatic urothelial carcinoma (IMvigor130): final overall survival analysis results from a randomised, controlled, phase 3 study. Lancet Oncol.

